# Teaching and learning how to make informed health choices: Protocol for a context analysis in Spanish primary schools

**DOI:** 10.12688/f1000research.51961.1

**Published:** 2021-04-22

**Authors:** Laura Martínez García, Laura Samsó Jofra, Pablo Alonso-Coello, Eukane Ansuategi, Laia Asso Mistral, Monica Ballesteros, Carlos Canelo-Aybar, Gonzalo Casino, Ana Gallego Iborra, Ena Pery Niño de Guzmán Quispe, Carolina Requeijo, Marta Roqué i Figuls, Karla Salas, Mar Ubeda, Iratxe Urreta, Sarah Rosenbaum

**Affiliations:** 1 Iberoamerican Cochrane Centre (IbCC) - Sant Pau Biomedical Research Institute (IIB-Sant Pau), Barcelona, Spain; 2 CIBER of Epidemiology and Public Health (CIBERESP), Barcelona, Spain; 3 Epidemiology and Public Health Department, Hospital de la Santa Creu i Sant Pau, Barcelona, Spain; 4 Osakidetza, OSI Donostialdea, University Hospital of Donostia, Library Service, Donostia, Spain; 5 Maternal and Child Health Service, General Subdirectorate of Health Promotion, Public Health Agency of Catalonia, Barcelona, Spain; 6 Andalusian Health Service, Malaga, Spain; 7 Health Services Research Group – Vall d’Hebron Research Institute (VHIR), Barcelona, Spain; 8 Vall d’Hebron University Hospital, Barcelona, Spain; 9 Clinical Epidemiology and Research Unit, University Hospital of Donostia, Donostia, Spain; 10 Centre for Informed Health Choices, Norwegian Institute of Public Health, Oslo, Norway

**Keywords:** Children’s health, critical thinking, evidence-based medicine, health education, health promotion, public health.

## Abstract

*Introduction*

The Informed Health Choices (IHC) project developed learning resources to teach primary school children (10 to 12-year-olds) to assess treatment claims and make informed health choices. The aim of our study is to explore the educational context for teaching and learning critical thinking about health in Spanish primary schools.

*Methods*

During the 2020-2021 school year, we will conduct 1) a systematic assessment of educational documents and resources, and 2) semi-structured interviews with key education and health stakeholders. In the systematic assessment of educational documents and resources, we will include state and autonomous communities’ curriculums, school educational projects, and commonly used textbooks and other health teaching materials. In the semi-structured interviews, we will involve education and health policy makers, developers of learning resources, developers of health promotion and educational interventions, head teachers, teachers, families, and paediatric primary care providers. We will design and pilot a data extraction form and a semi-structured interview guide to collect the data. We will perform a quantitative and a qualitative analysis of the data to explore how critical thinking about health is being taught and learned in Spanish primary schools.

*Conclusion*

We will identify opportunities for and barriers to teaching and learning critical thinking about health in Spanish primary schools. We will formulate recommendations—for both practice and research purposes—on how to use, adapt (if needed), and implement the IHC resources in this context.

## Introduction

People are constantly exposed to information about health. When people use unreliable information, they may harm their health or not consume their resources efficiently. ^1^ For this reason, people need to acquire health literacy (obtain, process, and understand health information) and think critically about health (use appropriate criteria to make judgements about health information). ^2- 4^ Therefore, they can assess the trustworthiness of health claims and make informed health decisions.

### The Informed Health Choices project

The Informed Health Choices (IHC) project aims to teach people to assess treatment claims and make informed health decisions. ^5^ As part of the IHC project, the IHC Working Group developed: 1) the IHC Key Concepts (list of concepts that individuals need to understand and apply when assessing claims about treatment effects and making health choices), ^6^ 2) the IHC resources (learning resources to teach children and their families to understand and apply some of the IHC key concepts), ^7- 9^ and 3) the CLAIM Evaluation Tools (database with questions to assess people’s understanding and ability to apply the IHC key concepts). ^10^

The IHC Working Group evaluated the effect of the IHC resources in a cluster randomised trial in Ugandan primary schools. ^11^ The study showed that the children (10 to 12-year-olds) who used the IHC resources improved their ability to assess treatment claims and retained this knowledge one year later. ^11, 12^

The IHC project has acquired greater relevance during the ongoing COVID-19 pandemic, considering that the current health situation is aggravated by an infodemic. The World Health Organization (WHO) defines “infodemic” as an excessive amount of information, in some cases correct and in others not, which makes it difficult for people to find reliable sources and guidance when they need them. ^13^ In this context, it is vital to teach people to critically assess health information (e.g., prolonged use of mask causes hypoxia) and to make informed health decisions (e.g., vaccination against COVID-19).

### Spanish education system

Spain is organized territorially into self-governing communities (17 autonomous communities and two autonomous cities), provinces, and municipalities. The Spanish education system follows a decentralised model where educational responsibilities are shared among all levels of government: state general authority (Ministry of Education), autonomous communities (Departments of Education), local authorities (Education Councils), and educational institutions ( Table 1). ^14, 15^

**Table 1.** Distribution of responsibilities between levels of government in Spanish education system.

Decision-making bodies	Distribution of responsibilities	Design of the basic curriculum
Ministry of Education and Vocational Training (MEFP)	- General organisation of the education system - Regulation of academic and professional titles, and basic rules for the development of the right to education - Establishment of the general plan for education - Evaluation and innovation of the learning integrated into the education system - Educational inspection - Design, planning and management of scholarships and financial support - Promotion of equality, non-discrimination, and universal accessibility policies within the scope of its powers - Management of the teaching staff policy and development of the foundations for the legal regime of public teaching service - Exercise of the functions of National Authority for the Erasmus+ Programme of the European Commission	- Establish the common contents and assessable learning standards of core subjects - Establish the minimum number of hours for core subjects (not be less than 50% of the total number of teaching hours generally established by each education authority) - Establish the assessable learning standards of specific subjects - Design the final evaluation for primary education, compulsory secondary education, and upper secondary education - Recognise the certificates awarded corresponding to regulated studies - Establish mixed curricula of the Spanish education system and other education systems - Promote actions to enhance the quality of educational institutions
Departments of Education of the autonomous communities	- Assume the regulations developed by the State rules - Assume the executive and administrative competences for managing the education system in the territory - Promote and strengthen education school autonomy - Evaluate school results and implement action plans	- Complement the contents of core subjects - Establish the contents of specific subjects and freely-structured subjects - Conduct methodological recommendations to educational institutions within the territory - Establish the teaching hours for all the subjects, with the exception of core subjects - Complement the evaluation criteria for the stage assessment - Establish assessment criteria and learning standards of the free subjects for the stage assessment - Expedite the certificates awarded corresponding to regulated studies - Promote actions to enhance the quality of education educational institutions
Education Councils	- Assume functions in areas that have a direct local impact	Without any responsibility on the design of the basic curriculum
Educational institutions	- Autonomy to develop, approve, and execute school educational projects, management projects, and organizational and functioning rules of the school.	- Complement the contents of all subjects on the basis of educational provision - Design and implement their own teaching and learning methods - Establish the number of hours for the different subjects

This table has been reproduced with permission from European Commission - Eurydice ^14^.

The legislative framework governing the Spanish education system is based on the Organic Law of Education, of 2006 ( *Ley Orgánica de Educación* - LOE), and the Organic Law for the Improvement of the Educational Quality, of 2013 ( *Ley Orgánica para la Mejora de la Calidad Educativa* - LOMCE). ^16, 17^ Currently there is a new Draft Organic Law of Modification of the LOE, of 2020 ( *Ley Orgánica de modificación de la LOE* - LOMLOE). ^18^ The Royal Decrees regulate the core curriculum of primary education, compulsory secondary education ( *Educación Secundaria Obligatoria*, ESO), and upper secondary education ( *Bachillerato)*. ^19, 20^

The Spanish education system is divided into four levels: 1) pre-primary education, organised into two cycles of three years (0-3 and 3-6 years old); 2) primary education (6-12 years old); 3) secondary education, organised into two cycles: compulsory secondary education (12-16 years old), and upper secondary education (16-18 years old) or vocational training; and 4) higher education, comprised of university or professional studies. ^21^ Basic education (primary and compulsory secondary education) is mandatory and free in schools supported with public funds. ^14^

In Spain there are three different types of schools according to their ownership and source of funding: 1) public schools, owned by the education authority and publicly-funded (Department of Education); 2) publicly-funded private schools, privately owned (educational institution) but publicly-funded (Departments of Education) through a regime of agreements; and 3) private schools, privately owned and privately-funded (educational institution). ^14^ In the school year 2020-2021, there are 14,151 schools that provide primary education; 75% public schools, 21% publicly-funded private schools, and 4% private schools. ^22^

The public educational expenditure in 2018 was 4.23% of the GDP (Gross Domestic Product), which was below the EU average (4.6%). ^23, 24^ The distribution of public expenditure was mainly among pre-primary and primary education (35%), and secondary education and vocational training (29.3%). ^24^

### Health promotion and educational interventions in Spanish schools

Health promotion interventions (interventions to enable people to increase control over and to improve their health) and health education interventions (interventions to improve people's health literacy) in schools have shown to improve the health of children and young people. ^25- 28^

Health promotion and education in schools requires intersectoral collaboration and partnerships between educational and health institutions. ^29^ In 1989, the Spanish Ministry of Education and the Ministry of Health signed a collaboration agreement to encourage the integration of health promotion and education in schools. ^30- 33^ In 1993, the country joined to European Network of Health Promoting Schools (ENHPS), which aims to integrate health promotion into every aspect of the curriculum, introduce healthy programmes and practices into schools’ daily routines, improve working conditions, and foster better relations both within the schools and between them and their local communities. ^33, 34^

The Spanish LOE educational law of 2006 defined two competences, “Knowledge and interaction with the physical world” and “Social and citizenship” that included health promotion and education (essential knowledge, skills, and attitudes for participating in society) directly and indirectly, respectively. ^35^ However, the current Spanish LOMCE educational law of 2013 includes health competencies in a transversal way, and its contents are distributed among several knowledge areas (Biology, Physical Education, and Ethical values/Education for citizenship). ^35^

In Spain, the schools have the ultimate responsibility to integrate health promotion and educational interventions into their educational projects. ^32^ This means to foster the value of health among all different members of the school community, throughout the school year, in order to facilitate healthy behaviours, promote autonomous decision-making and personal choices of healthy lifestyles, and establish long-term positive attitudes towards health care. ^32^

### Contextualization of the Informed Health Choices resources in Spanish primary schools

The contextualization of the IHC resources comprises activities to explore how these resources can be used in a different context from the one that they were originally designed for (primary schools in Uganda). These activities may include, for example: 1) context analysis to explore conditions for teaching critical thinking about health, 2) translation of the IHC resources, 3) pilot testing of the IHC resources, 4) adaptation of the IHC resources (if needed), 5) assessment of the effects of using the IHC resources, or 6) translation and validation of the CLAIM Evaluation Tools. ^36- 38^

**Figure 1.**
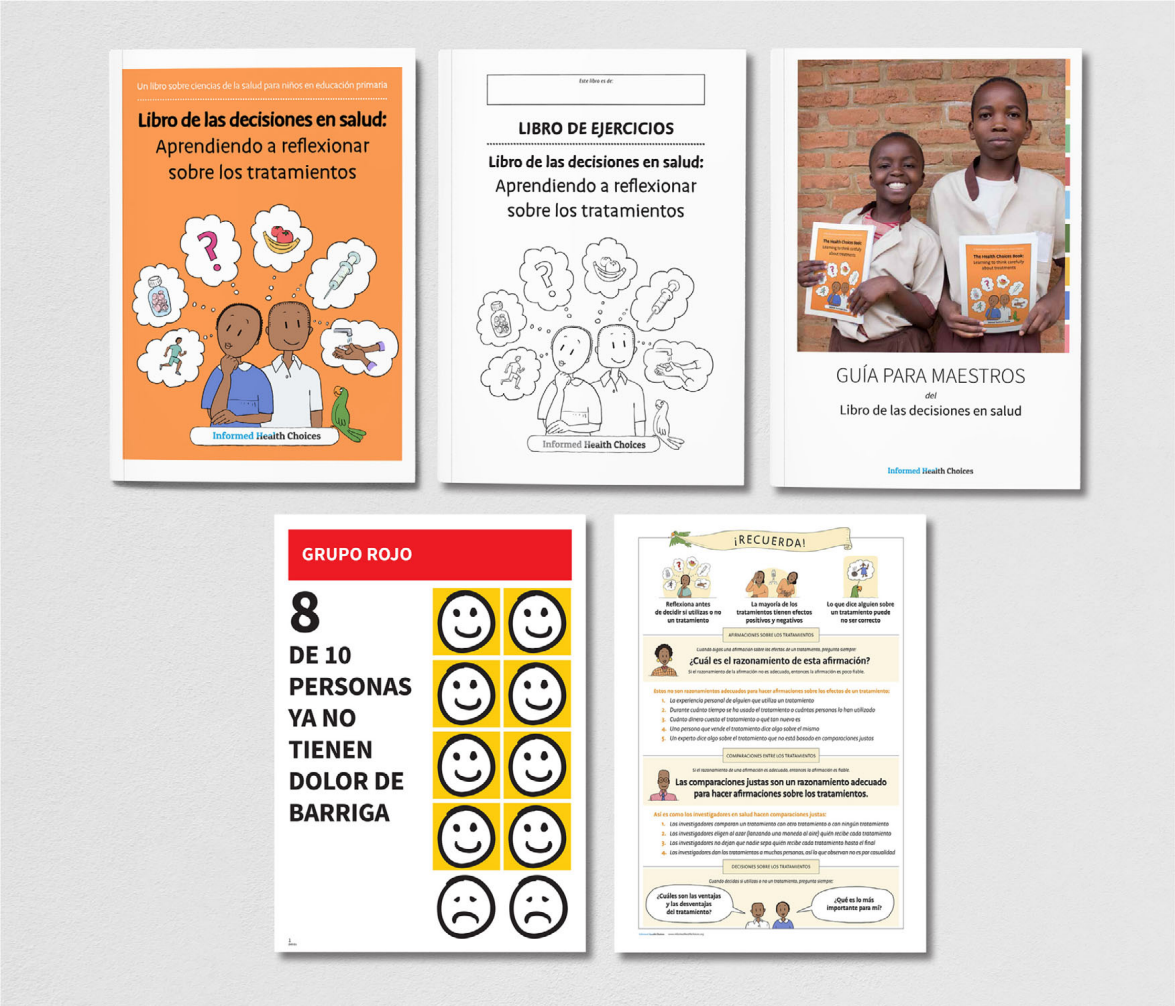
Informed Health Choices learning resources for primary school children (Spanish translation).

The IHC resources have already been translated into Spanish ( Figure 1), and a pilot study is being conducted in schools in Barcelona to explore the students and teachers’ experience when using the IHC resources. ^39- 42^ The next step is to analyse the educational context to ensure the relevance and appropriateness of the IHC primary school resources for Spanish primary schools.

## Objectives

### Primary objective

To explore the educational context for teaching and learning critical thinking about health in Spanish primary schools.

**Secondary objectives**

• To identify and describe relevant educational documents and resources that support teaching and learning of critical thinking about health, and that are available in Spanish primary schools.
• To explore the experience and perspective of key education and health stakeholders regarding teaching and learning critical thinking about health in Spanish primary schools.
• To identify factors that can potentially impact the implementation of the IHC resources in Spanish primary schools.

## Methods

During the 2020-2021 school year, we will conduct 1) a systematic assessment of educational documents and resources, and 2) semi-structured interviews with key education and health stakeholders; based on methods proposed by the IHC Working Group. ^43^ Table 2 describes the different steps of the study. We will report qualitative findings using the COREQ (Consolidated criteria for reporting qualitative research) checklist. ^44^

**Table 2.** Tasks of the study.

Tasks		Participants	Activities
**1. Protocol**			
	1. 1. Development of the protocol	Researchers	- Develop the study protocol - Request the approval of Ethics Committee
	1.2. Publication of the protocol	Researchers	- Submit the manuscript to a peer-reviewed journal
**2. Systematic assessment of educational documents and resources**			
	2.1. Documents identification	Researchers Head teachers	- Identify the state and autonomous communities’ curriculums - Identify school educational projects (approx. 17) - Identify commonly used textbooks and other health teaching materials
	2.2. Documents selection	Researchers	- Screen titles and full texts - Cross-check the selection
	2.3. Data collection - Data extraction form	Researchers	- Design, pilot, and refine a data extraction form - Data collection - Cross-check the data
**3. Semi-structured interviews with key education and health stakeholders**			
	3.1. Participants identification	Researchers	- Identify key education and health stakeholders (approx. 36) - Contact, inform, and invite potential participants - Request written informed consent and declare potential conflicts of interest
	3.2. Data collection - Semi-structured interviews	Researchers Key education and health stakeholders	- Design, pilot, and refine a semi-structured interview guide - Develop a training video to present the IHC project - Conduct the interviews - Audio record and transcribe interviews - Send interview transcripts for approval - Anonymise the data
**4. Data analysis**			
	4.1. Quantitative analysis	Researchers	- Descriptive analysis
	4.2. Qualitative analysis	Researchers	- Descriptive thematic synthesis - Map IHC Key Concepts - Cross-check the analysis - Summarise the data
**5 Dissemination of the results**			
	5.1. Publication in a peer-reviewed journal	Researchers	- Draft the manuscript - Submit the manuscript to a peer-reviewed journal
	5.2. Online communication	Researchers	- Online communication via related websites, electronic bulletins, and social media
	5.3. Tailored presentations	Researchers	- Tailor presentations for key education and health stakeholders

### Systematic assessment of educational documents and resources

Eligibility criteria

We will include educational documents and resources (state and autonomous communities curriculums, school educational projects, textbooks and other health teaching materials) that cover aspects related to critical thinking about health (critical thinking in general, health in general, and critical thinking specifically about health), focused on primary education, available in the Spanish context, written in any official or co-official language of the country (Spanish, Catalan, Galician, Valencian, or Basque), and currently used during 2020-2021 school year.

Information sources and search strategy

To identify the state and autonomous communities’ curriculums, we will conduct a manual search on the website of the Spanish Ministry of Education and Vocational Training, ^45^ as well as on the websites of the corresponding departments of the autonomous communities.

To identify school educational projects, we will select a convenience sample of schools from the Spanish Ministry of Education registry. ^22^ We will aim for representativeness of schools based on geographic area (autonomous communities), and source of funding of schools (public, publicly-funded private, or private) ( Table 3). We expect to include a sample of approximately 34 schools. We will contact, inform, and invite head teachers from selected schools (invitation e-mail, first e-mail reminder, second e-mail reminder, and telephone reminder) ( *Extended data 1* ^46^). If a school does not respond or does not agree to participate, we will select the next eligible school from the registry.

**Table 3.** Stratified sampling strategy.

Strata		Expected sample of schools	Expected sample of participants
**Strata 1 - Geographic area (autonomous communities)**			
	Andalucía	2	2
	Aragón	2	2
	Principado de Asturias	2	2
	Illes Balears	2	2
	Canarias	2	2
	Cantabria	2	2
	Castilla y León	2	2
	Castilla-La Mancha	2	2
	Cataluña	2	2
	Comunitat Valenciana	2	2
	Extremadura	2	2
	Galicia	2	2
	Comunidad de Madrid	2	2
	Región de Murcia	2	2
	Comunidad Foral de Navarra	2	2
	País Vasco	2	2
	La Rioja	2	2
	**Total**	**34**	**34**
**Strata 2- Source of funding of school**			
	Public schools	17	6 *
	Publicly-funded private schools or private schools	17	6 *
	**Total**	**34**	12*
**Strata 3 - Participant profile**			
**System level**			
	Education policy makers	-	4
	Health policy makers	-	4
	Developers of learning resources	-	4
	Developers of health promotion and educational interventions	-	4
**School level**			
	Head teachers	-	4
	Teachers	-	4
	Families	-	4
**Health care level**			
	Physicians	-	4
	Nurse practitioners	-	4
	**Total**		**36**

* We will consider strata 2 only for head teachers, teachers, and families’ profiles.

To identify commonly used textbooks and other health teaching materials, we will ask head teachers and teachers from the participating schools for suggestions.

Document selection

One author will screen titles and full texts to identify potentially eligible documents for inclusion. A second author will cross-check the selection. The two authors will resolve potential disagreements by discussion, and if necessary, by consulting a third author.

Data collection

We will design, pilot and refine a data extraction form that will include the following information: 1) document identification, 2) description of the document, 3) description of the content related to critical thinking, health, and critical thinking about health, and 4) mapping of the content with IHC Key Concepts (if applicable) ( *Extended data 2* ^46^).

One author will perform the data collection, and a second author will cross-check the data. The two authors will resolve potential disagreements by discussion, and if necessary, by consulting a third author.

### Semi-structured interviews with key education and health stakeholders

Participants

To cover key education and health stakeholders, we will involve education and health policy makers, developers of learning resources, developers of health promotion and educational interventions, head teachers, teachers, families, and paediatric primary care providers (physicians and nurse practitioners). We will identify participants from 1) articles included in the systematic assessment of educational documents and resources, 2) participating schools included in the systematic assessment, and 3) expert colleagues. We will aim for representativeness of participants based on geographic area (autonomous communities), source of funding of schools (public, publicly-funded private, or private), and profile of participants (education and health policy makers, developers of learning resources, developers of health promotion and educational interventions, head teachers, teachers, families, physicians, and nurse practitioners) ( Table 3). We expect to include a sample of approximately 36 participants, although we will continue recruiting and collecting data until information becomes repetitive and no new information emerges (sampling saturation). ^47, 48^

We will contact, inform, and invite potential participants (invitation e-mail, first e-mail reminder, second e-mail reminder, and telephone reminder) ( *Extended data 1* ^46^). Those who agree to participate will be asked to complete a written informed consent ( *Extended data 3* ^46^) and declare potential conflicts of interest. ^49^

Data collection

We will design, pilot and refine a semi-structured interview guide that will include the following information: 1) participant identification, 2) description of the participant (age, gender, profile, working institution, and autonomous community), 3) participant’s experience on how critical thinking about health is being taught and learned in Spanish primary schools (curriculum, subjects, educational documents and resources, and evaluation), 4) participant’s perspective on the relevance of teaching and learning critical thinking about health in Spanish primary schools (relevance in the educational context), 5) participant’s perspective on how to implement IHC resources in Spanish primary schools (potential facilitators and barriers ^50^) ( *Extended data 4* ^46^).

Before each interview, we will introduce the participants to the IHC project, the IHC resources, and the pilot study in Barcelona with a training video. ^5, 7- 9, 42^ After that, one trained researcher will conduct the interviews face to face or via teleconference. Each interview will last approximately one hour and will be audio recorded and transcribed. The interview transcripts will be sent to participants for approval before conducting the data analysis.

### Data analysis

Quantitative analysis

We will perform a descriptive analysis of the categorical variables (absolute and relative frequencies), and the continuous variables (median and range) ( *Extended data 5* ^46^).

Qualitative analysis

We will register in an Excel sheet quotes from: 1) educational documents and resources, and 2) semi-structured interviews. We will identify themes related to the educational context applying a three-step descriptive thematic synthesis: 1) codifying extracted quotes, 2) proposing descriptive themes, and 3) identifying main themes based on conceptual similarities within and across quotes. ^51^ If applicable, we will map the extent to which the educational documents and resources overlap with the IHC Key Concepts through a data matrix, including documents as rows and the IHC Key Concepts as columns. One author will conduct the analysis, and a second author will cross-check it. The two authors will resolve potential disagreements by discussion, and if necessary, by consulting a third author.

Finally, using the summarised data, we will explore the nature of the phenomena (how critical thinking about health is being taught and learned in Spanish primary schools), and the possible explanations for the findings. Furthermore, we will deepen our understanding of the opportunities for and barriers to teaching and learning critical thinking in general, about health in general, and critical thinking specifically about health.

### Dissemination of the results

The dissemination activities of the study results will include: 1) publication in a peer-reviewed journal; 2) online communication via related websites, electronic bulletins, and social media; and 3) tailored presentations for key education and health stakeholders.

### Ethical considerations

The study protocol has obtained an approval exemption (does not include patients, biological specimens, or clinical data) from the Ethics Committee of the Hospital de la Santa Creu i Sant Pau (Barcelona, Spain).

We will inform participants about the study and request their written informed consent and declaration of potential conflicts of interest. We will not collect any sensitive personal data (racial or ethnic origin, political opinions, religious or philosophical beliefs, trade-union membership, genetic data, biometric data, health-related data, or data concerning a person’s sex life or sexual orientation). ^52^ We will anonymise personal data, coding the name of the participants and the institutions. Only researchers will have access to the identifier list (with the code linked to personal data). Personal data will be deleted five years after the study has concluded.

### Study status

**Figure 2.**
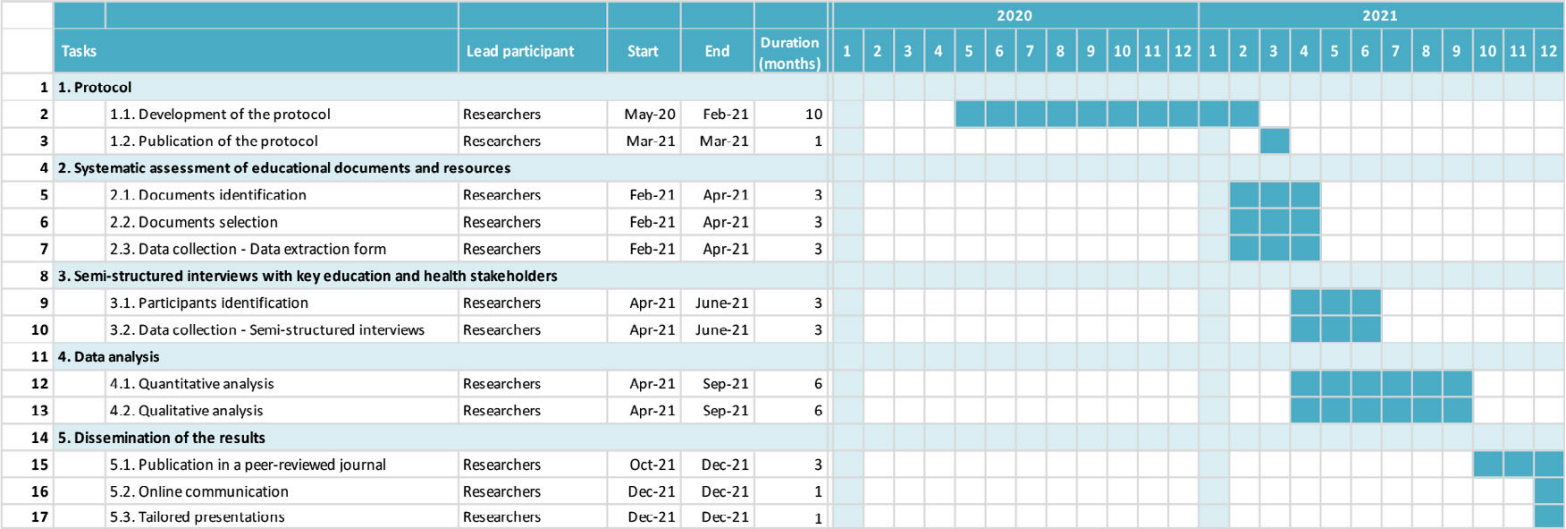
Gantt chart of the study.

Figure 2 is a Gantt chart illustrating the schedule of the context analysis. To date, we started the systematic assessment of relevant education documents and resources.

## Discussion

People need to learn to think critically about health and make informed health decisions. The IHC project proposed to start this challenge by teaching children and using the IHC resources, which were specifically designed and evaluated to achieve this goal. The next step is to support the dissemination of the IHC resources, thus help to empower people around the world to make well-informed decisions.

### Our study in the context of current knowledge

The context analysis is an important step to complete before developing innovative health promotion and educational interventions in schools, such as the IHC resources. This analysis can identify factors that might affect scaling up at a stage that is early enough to inform the development of the interventions.

During the trial to evaluate the effects of the IHC resources in primary schools in Uganda, the IHC Working Group conducted a process evaluation to identify factors affecting their implementation. ^11, 49^ This study showed that participants valued the IHC resources, although they highlighted the need to incorporate the lessons into the national curriculum to scale up their use. ^49^ They also found that the cost of the IHC resources was a critical barrier to scale up their use. ^49^ After this experience, they conducted a context analysis before developing the IHC resources for secondary schools. ^43^ Therefore, they are designing the resources considering relevant factors from the context of reference. ^53^

Lund *et al*. 2018 conducted a market analysis to explore the demand, adequacy for the curriculum, and market conditions for introducing the IHC resources in Norwegian primary and secondary schools. ^54^ They analysed key documents and interviewed teachers and other key stakeholders. ^54^ One of the primary findings was that teaching critical thinking about health fits into the curriculum and should be prioritised; however, classroom time is limited and critical thinking about health cuts across subjects. ^54^ The teachers who participated pointed out that they are empowered to decide what to teach, how, and with what learning resources. ^54^ Further work is needed to adapt the IHC resources (e.g., use as little classroom time as possible, facilitate collaboration across subjects and grades, and engage teachers in the design) and scale up its use in Norwegian primary and secondary schools.

### Strengths and limitations of the study

Our proposal has several strengths. We are building on previous studies and using multiple methods and triangulation to ensure the trustworthiness of our findings. ^43, 54^ Furthermore, this study is part of a comprehensive project of contextualization activities that we have completed (translation of the IHC resources) or that are ongoing (pilot study) to explore how Spanish primary schools can benefit from the IHC resources. ^39- 42^

Our proposal also has some limitations. We will face numerous challenges, as we will have to consider different educational contexts and languages (autonomous communities) within the same country (Spain). In addition, the ongoing COVID-19 pandemic may be a significant barrier for the recruitment of participants.

### Implications for practice and research

We will formulate recommendations—for both practice and research purposes—on how to use, adapt (if needed), and implement the IHC resources in Spanish primary schools. The findings of the contextualization activities will inform the design of a cluster randomised trial to determine the effectiveness of the IHC resources in this context prior to scaling up their use.

## Data availability

### Underlying data

No data are associated with this article.

### Extended data

Figshare: IHC@BCNContextAnalysis. https://doi.org/10.6084/m9.figshare.14152880. ^46^

This project contains the following extended data:

- Extended data 1 – Information for schools and participants (documents available in Spanish)
- Extended data 2 – Data extraction form for educational documents and resources
- Extended data 3 – Written informed consent form for participants
- Extended data 4 – Guide for the semi-structured interviews
- Extended data 5 – Descriptive-quantitative variables of the study

Data are available under the terms of the Creative Commons Attribution 4.0 International license (CC-BY 4.0).
