# Supplementary material for: Teaching and learning how to make informed health choices: Protocol for a context analysis in Spanish primary schools
Source: F1000Res. 2021 Aug 11;10:312. Originally published 2021 Apr 22. [Version 2] doi: 10.12688/f1000research.51961.2 (PMC8474100; doi:10.12688/f1000research.51961.2)
Supplement: Peer review history

# Peer review: Reviewer response for version 2

**DOI:** 10.5256/f1000research.70813.r91637

**Published:** 2021-09-23

**Authors:** Loai Albarqouni

**Affiliations:** 1 The Institute for Evidence-Based Healthcare, Faculty of Health Sciences and Medicine, Bond University, Gold Coast, Qld, Australia

I would like to thank the authors for responding to all my comments.

Is the study design appropriate for the research question?

Yes

Is the rationale for, and objectives of, the study clearly described?

Yes

Are sufficient details of the methods provided to allow replication by others?

Partly

Are the datasets clearly presented in a useable and accessible format?

Not applicable

Reviewer Expertise:

Evidence-based practice

I confirm that I have read this submission and believe that I have an appropriate level of expertise to confirm that it is of an acceptable scientific standard.

# Peer review: Reviewer response for version 1

**DOI:** 10.5256/f1000research.55177.r88339

**Published:** 2021-07-22

**Authors:** Elaine M. Finucane

**Affiliations:** 1 Health Research Board-Trials Methodology Research Network (HRB-TMRN), Evidence Synthesis Ireland and Cochrane Ireland, School of Nursing & Midwifery, NUI Galway, Galway, Ireland

Well done on a very timely study whose primary aim is to explore the educational context for teaching and learning critical thinking about health in Spanish primary schools. To achieve this, the authors plan to conduct a systematic assessment of educational documents and resources and conduct semi-structured interviews with key education and health stakeholders.

 I have just a few comments:

**Introduction:**

** ‘***In this context, it is vital to teach people to critically assess health information (e.g., prolonged use of mask causes hypoxia) and to make informed health decisions (e.g., vaccination against COVID-19).' - *I feel the context of the examples used in this sentence need to be clearer.

**Methods:**

*‘In the semi-structured interviews, we willinvolve education and health policy makers, developers of learning resources, developers of health promotion and educational interventions, head teachers, teachers, families, and paediatric primary care providers.' - *Where the authors state that families will be participants, I think it would be helpful if it were clearly stated if this included children as stakeholders also.

 Involving stakeholders, including service users, in all stages of a study, from design through to dissemination supports relevance, diversity, and accessibility, which would seem particularly relevant to this study as the authors note it will face challenges as they ‘ *will have to consider different educational contexts and languages (autonomous communities) within the same country (Spain)*’. To this end, I am surprised not to see a PPI group (public and patient involvement in research) actively involved throughout the different study processes.  

 I wish the authors all the best with their study.

Is the study design appropriate for the research question?

Yes

Is the rationale for, and objectives of, the study clearly described?

Yes

Are sufficient details of the methods provided to allow replication by others?

Yes

Are the datasets clearly presented in a useable and accessible format?

Not applicable

Reviewer Expertise:

Trial Methodology & Evidence Synthesis.

I confirm that I have read this submission and believe that I have an appropriate level of expertise to confirm that it is of an acceptable scientific standard.

# Response

**Published:** 2021-08-06

**Authors:** Laura Martínez García

**Affiliations:** Iberoamerican Cochrane Centre - Sant Pau Biomedical Research Institute (IIB-Sant Pau), Spain

We would like to thank Dr Finucane for her time and effort providing feedback on our manuscript. Below we describe how we have addressed each of her comments about the manuscript.

**Comment 1 – COVID-19 examples**

**Response 1**

 We amended the text in Introduction section according to the reviewer’s suggestion. The text now reads: *‘In this context, it is vital to teach people to critically assess health information (e.g., how to assess the reliability of the claim ‘If you wear a face mask for a long time, you may have hypoxia’) and to make informed health decisions (e.g., how to decide whether to vaccinate against COVID-19).'*

**Comment 2 – Participants**

**Response 2**

 The perspective of children is considered in our previous study *‘Learning to make informed health choices: Protocol for a pilot study in schools in Barcelona’ *[1]. The aim of this study is to explore both the students’ and teachers’ experience when using the IHC resources in the context of Barcelona (Spain). However, the present study is more related to education and health policies. Therefore, we decided not to include young children as a target stakeholder.

 To clarify this issue, we amended the text in Methods section according to the reviewer’s suggestion. The text now reads: *‘To cover key education and health stakeholders, we will involve education and health policy makers, developers of learning resources, developers of health promotion and educational interventions, head teachers, teachers, families (without including children), and paediatric primary care providers (physicians and nurse practitioners)’.*

**Comment 3 – Public and Patient Involvement group**

**Response 3**

 As far as we know, there are no established PPI groups - as in the health area - for the education area. We would be grateful to the reviewer to suggest any interest group for considering in our next studies.

**References**

 1. Martínez García L, Alonso-Coello P, Asso Ministral L, et al. Learning to make informed health choices: Protocol for a pilot study in schools in Barcelona. F1000Res. 2019; 8: 2018.

# Peer review: Reviewer response for version 1

**DOI:** 10.5256/f1000research.55177.r84053

**Published:** 2021-06-28

**Authors:** Loai Albarqouni

**Affiliations:** 1 The Institute for Evidence-Based Healthcare, Faculty of Health Sciences and Medicine, Bond University, Gold Coast, Qld, Australia

Thanks for inviting me to review this protocol for an interesting research study. The authors aim to explore the educational context for teaching and learning critical thinking about health in Spanish primary schools. To achieve these aims, the authors plan to conduct a systematic assessment of educational resources and interviews with relevant stakeholders.

**Comments:**

- Authors will select a convenience sample of 34 schools. It would be good if authors can put this in context e.g. the total number of schools (population).
- I wonder if headteachers are easily identified and if they are the best to address their queries. I also wonder if they should have a unified approach for their request to the documents from the headteachers.
- Authors might consider describing their selection criteria e.g. would they include a document that has just marginally covered a few aspects relevant to critical thinking about health.
- Authors might consider describing their qualitative analysis approach e.g. thematic analysis.
- Similar authors might consider describing their approach of dealing with duplicating or overlapping aspects identified in the educational resources.

Is the study design appropriate for the research question?

Yes

Is the rationale for, and objectives of, the study clearly described?

Yes

Are sufficient details of the methods provided to allow replication by others?

Partly

Are the datasets clearly presented in a useable and accessible format?

Not applicable

Reviewer Expertise:

Evidence-based practice

I confirm that I have read this submission and believe that I have an appropriate level of expertise to confirm that it is of an acceptable scientific standard, however I have significant reservations, as outlined above.

# Response

**Published:** 2021-08-06

**Authors:** Laura Martínez García

**Affiliations:** Iberoamerican Cochrane Centre - Sant Pau Biomedical Research Institute (IIB-Sant Pau), Spain

We would like to thank Dr Albarqouni for his time and effort providing feedback on our manuscript. Below we describe how we have addressed each of the comments about the manuscript.

**Comment 1 – Sample**

**Response 1**

 The context of the study was described in the Introduction section ‘Spanish education system’. In this section we included the number of schools in the country, the text read: *‘In the school year 2020-2021, there are 14,151 schools that provide primary education; 75% public schools, 21% publicly-funded private schools, and 4% private schools.’*

**Comment 2 – Head teachers**

**Response 2**

 The Spanish Ministry of Education registry ( https://www.educacion.gob.es/centros/home.do) included the following information for each school: 1) identification (including telephone number, email, and web page), 2) situation, and 3) source of funding (public schools, publicly-funded private schools, and private schools). Based on this information, we will be able to contact the selected schools and their headteachers directly. We described the recruitment strategy in the Methods section, the text read: *‘We will contact, inform, and invite head teachers from selected schools (invitation e-mail, first e-mail reminder, second e-mail reminder, and telephone reminder) (Extended data 1)’. Furthermore, we developed an informative document to introduce the study to head teachers (‘Extended data 1 – Information for schools and participants’*, ( https://figshare.com/articles/online_resource/IHC_BCNContextAnalysis/14152880).

 We will have to wait for the findings of the study to assess whether 1) the recruitment strategy is efficient, and 2) the request should be addressed by head teachers.

**Comment 3 – Document selection criteria**

**Response 3**

 The selection criteria were described in Methods section ‘Eligibility criteria’, which stated:

- *Educational documents and resources (state and autonomous communities curriculums, school educational projects, textbooks and other health teaching materials)*
- *Covered aspects related to critical thinking about health (critical thinking in general, health in general, and critical thinking specifically about health)*
- *Focused on primary education*
- *Available in the Spanish context*
- *Written in any official or co-official language of the country (Spanish, Catalan, Galician, Valencian, or Basque)*
- *Currently used during 2020-2021 school year*

We will have to wait for the findings of the study to describe in depth the aspects covered by identified educational documents and resources.

**Comment 4 and 5 – Qualitative analysis**

**Response 4 and 5**

 We amended the text in Methods section ‘Qualitative analysis’ according to the reviewer’s suggestion. The text now reads: *‘We will analyse and synthesise qualitative data using a thematic synthesis. We will register in an Excel sheet quotes from: 1) educational documents and resources, and 2) semi-structured interviews. We will identify themes related to the educational context applying a three-step descriptive thematic synthesis: 1) codifying extracted quotes, 2) proposing descriptive themes, and 3) identifying main themes based on conceptual similarities within and across quotes [51]. We will describe the extent of duplication and overlapping themes within and across documents. If applicable, we will map how themes reflect the IHC Key Concepts framework through a data matrix (including documents as rows and the IHC Key Concepts as columns) [6]. One author will codify extracted quotes and propose descriptive themes. Two authors will select the descriptive themes, identify main themes, and assess the overlap with the IHC Key Concepts guided by iterative discussion, and if necessary, by consulting a third author. The authors' team will approve the final synthesis of findings.’*

**References**

 6. Oxman AD, Chalmers I, Austvoll-Dahlgren A, et al.: Key Concepts for assessing claims about treatment effects and making well informed treatment choices [version 2; peer review: 3 approved]. F1000Res. 2019; 7: 1784.

 51. Thomas JHA, Synthesis NM: Combining results systematically and appropriately. In: Gough D, Oliver S, Thomas J, editor. An Introduction to Systematic Reviews. London: Sage Publications Ltd; 2012.
